# Antithrombotic therapy in high-risk patients after percutaneous coronary intervention; study design, cohort profile and incidence of adverse events

**DOI:** 10.1007/s12471-021-01606-2

**Published:** 2021-09-01

**Authors:** R. H. Olie, P. E. J. van der Meijden, M. J. A. Vries, L. Veenstra, A. W. J. van ‘t Hof, J. M. ten Berg, Y. M. C. Henskens, H. ten Cate

**Affiliations:** 1 Thrombosis Expertise Centre, Heart and Vascular Centre, Department of Internal Medicine, Maastricht University Medical Centre (MUMC+), Maastricht, The Netherlands; 2 Cardiovascular Research Institute Maastricht (CARIM), Maastricht University, Maastricht, The Netherlands; 3 Department of Cardiology, MUMC+, Maastricht, The Netherlands; 4 Department of Cardiology, St Antonius Hospital, Nieuwegein, The Netherlands; 5 Central Diagnostic Laboratory, MUMC+, Maastricht, The Netherlands; 6 Department of Cardiology, Zuyderland Medical Centre, Heerlen, The Netherlands

**Keywords:** Anticoagulation, Antiplatelet therapy, Antithrombotic treatment, Percutaneous coronary intervention, Coronary artery disease, Bleeding

## Abstract

**Background:**

Patients with multiple clinical risk factors are a complex group in whom both bleeding and recurrent ischaemic events often occur during treatment with dual/triple antithrombotic therapy after percutaneous coronary intervention. Decisions on optimal antithrombotic treatment in these patients are challenging and not supported by clear guideline recommendations. A prospective observational cohort study was set up to evaluate patient-related factors, platelet reactivity, genetics, and a broad spectrum of biomarkers in predicting adverse events in these high-risk patients. Aim of the current paper is to present the study design, with a detailed description of the cohort as a whole, and evaluation of bleeding and ischaemic outcomes during follow-up, thereby facilitating future research questions focusing on specific data provided by the cohort.

**Methods:**

We included patients with ≥ 3 predefined risk factors who were treated with dual/triple antithrombotic therapy following PCI. We performed a wide range of haemostatic tests and collected all ischaemic and bleeding events during 6–12 months follow-up.

**Results:**

We included 524 high-risk patients who underwent PCI within the previous 1–2 months. All patients used a P2Y12 inhibitor (clopidogrel *n* = 388, prasugrel *n* = 61, ticagrelor *n* = 75) in combination with aspirin (*n* = 397) and/or anticoagulants (*n* = 160). Bleeding events were reported by 254 patients (48.5%), necessitating intervention or hospital admission in 92 patients (17.5%). Major adverse cardiovascular events (myocardial infarction, stroke, death) occurred in 69 patients (13.2%).

**Conclusion:**

The high risk for both bleeding and ischaemic events in this cohort of patients with multiple clinical risk factors illustrates the challenges that the cardiologist faces to make a balanced decision on the optimal treatment strategy. This cohort will serve to answer several future research questions about the optimal management of these patients on dual/triple antithrombotic therapy, and the possible value of a wide range of laboratory tests to guide these decisions.

**Supplementary Information:**

The online version of this article (10.1007/s12471-021-01606-2) contains supplementary material, which is available to authorized users.

## What’s new?

- Increasingly, complex patients are treated with percutaneous coronary intervention (PCI), leading to a group of high-risk patients with multiple clinical risk factors being treated with dual or even triple antithrombotic therapy following PCI.
- These complex patients are frequently underrepresented in large clinical trials, and thus there is little evidence on optimal treatment.
- This cohort study was designed to evaluate patient-related factors, residual platelet reactivity, a broad spectrum of biomarkers, and bleeding questionnaires in predicting adverse events.
- Almost half of patients had at least one bleeding event during 6–12 months of dual/triple antithrombotic therapy and in 13.2% major adverse cardiovascular events occurred.

## Introduction

Percutaneous coronary intervention (PCI) is the treatment of choice in most patients with acute coronary syndrome (ACS) and frequently performed in patients with chronic coronary artery syndrome [1]. As results with PCI have improved due to better stents and antithrombotic treatment, increasingly complex patient populations are treated. International guidelines recommend a period of 6–12 months of dual antiplatelet therapy (DAPT) after PCI, sometimes in combination with oral anticoagulation if other comorbidities (e.g. atrial fibrillation) demand to do so [1, 2]. Thus, cardiologists are more and more challenged in treating complex, high-risk patients with dual or triple antithrombotic therapy. With the introduction of the more potent P2Y12 inhibitors prasugrel and ticagrelor next to clopidogrel [3, 4], and the widespread availability of direct oral anticoagulants (DOACs) next to vitamin K antagonists (VKAs), physicians are enabled to select different and individualised treatment regimens. Although evidence on optimal treatment exists for most patients, “high-risk” patients with multiple clinical risk factors (in whom both bleeding complications and recurrent ischaemic events occur more often) remain a challenging group. However, these patients are frequently excluded from or underrepresented in the large clinical trials, and although several bleeding risk scores have been developed, these scores have not been specifically validated in high-risk subjects [5].

This cohort study was designed to provide evidence on predictors, safety and outcome in a relevant subgroup of high-risk patients, and is part of an ongoing clinical care pathway. Patients are managed based on current international guidelines during the 6–12 month period of combined antithrombotic treatment following PCI (either with ACS indication or elective procedure). The clinical care pathway involves the assessment of the risk balance between thrombosis and bleeding prevention by identification and, if possible, removing such risk enhancing factors. In this study, we aim to evaluate patient-related factors, on-treatment platelet reactivity, biomarkers and bleeding questionnaires in predicting adverse events in high-risk patients. Future goals are to optimise the therapeutic windows of platelet functions tests (PFTs) for this specific group and to validate and/or develop risk estimation tools for prediction of bleeding complications in a population with multiple clinical risk factors.

The aim of the current cohort profile paper is to present a detailed description of the cohort as a whole, with evaluation of bleeding and ischaemic outcomes during follow-up, thereby facilitating future research questions focusing on specific data provided by the cohort.

## Methods

This prospective observational cohort study is conducted at the Thrombosis Expertise Centre in the Maastricht University Medical Centre (MUMC+) in the Netherlands. The medical ethics committee (METC) of the MUMC+ approved this study as an evaluation of patient care analysis (NL38767.068.11, METC number 11-2-096), and all patients provided written informed consent.

### Study population

Patients treated with PCI or coronary thrombolysis between May 2014 and May 2019 were screened for the presence of 3 or more predefined risk factors (Tab. 1) by one dedicated interventional cardiologist. These patients, all being treated with either DAPT or a combination of antiplatelet therapy with oral anticoagulants, were referred to a specialised outpatient clinic within the Thrombosis Expertise Centre for assessment of their bleeding risks and ischaemic risks. After informed consent was obtained, data on patient history, medication and comorbidities were collected, and blood was drawn for extensive haemostatic and genetic testing. Treatment decisions and subsequent medication switches were not part of the study, and initiated on the treating physician’s discretion, although all this information on medication switches was collected in the dataset.

**Table 1:** Inclusion and exclusion criteria

**Inclusion criteria**	**Definition**
*PCI 30–90 days before study inclusion*	Elective or emergency procedure
*Dual/triple antithrombotic therapy*	Including a P2Y12 inhibitor
*Classified as ‘vulnerable’ by ≥* *3* *predefined risk factors:*	Age ≥ 75 years
	Female gender
	Renal dysfunction (MDRD-eGFR ≤ 60 ml/min)
	Body weight ≤ 60 kg
	Hypertension (previously diagnosed, or on medication)
	Diabetes mellitus
	Anaemia (Hb < 8.2 mmol/l for men, < 7.3 mmol/l for women)
	Previous stroke
	Previous major bleeding
	Liver dysfunction (known hepatitis or transplant)
	History of gastric/duodenal ulcers
	Daily use of NSAIDs or SSRIs
	Triple antithrombotic therapy (DAPT + oral anticoagulants)
	Previous in-stent thrombosis or high risk coronary stent (≥ 3 lesions treated, total stent length > 60 mm, last remaining vessel, or left main coronary artery stenting)
**Exclusion criteria**	**Definition**
*Known platelet function disorders*	Previously diagnosed platelet function disorders
*Recent coronary intervention*	PCI or CABG ≤ 7 days
*Recent new ischaemic event*	ACS or stroke ≤ 7 days
*Signs of active infection*	Fever, antibiotic treatment or hospital admission during laboratory assessment of platelet function
*Medication non-compliance*	Confirmed non-compliance in antithrombotic medication by patient interview or pharmacy dispensing

*PCI* percutaneous coronary intervention,* MDRD-eGFR* Modification of Diet in Renal Disease—estimated glomerular filtration rate,* Hb* Haemoglobin,* NSAIDs* non-steroidal anti-inflammatory drugs,* SSRIs* selective serotonin reuptake inhibitors,* DAPT* dual antiplatelet therapy,* CABG* coronary artery bypass graft*, ACS* acute coronary syndrome

### Clinical care pathway

**Fig. 1:**
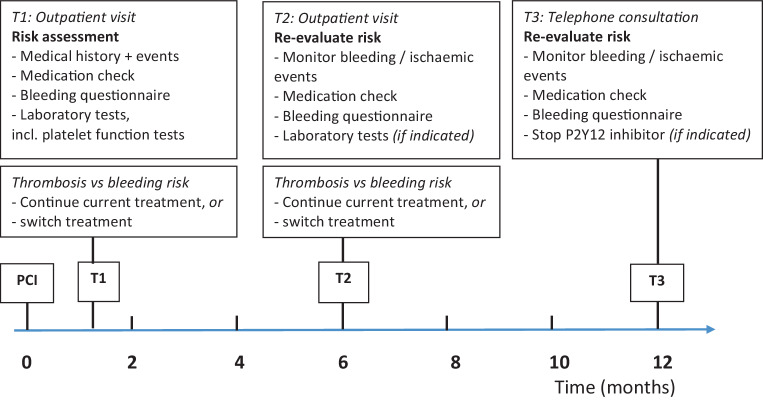
Timeline of the clinical care pathway

The clinical care pathway is illustrated in Fig. 1. At the first visit (1–2 months after PCI) information on medical history, medication and compliance was collected. A thorough history on both previous and current minor and major bleeding events was taken, using the International Society on Thrombosis and Haemostasis Bleeding Assessment Tool (ISTH-BAT) [6]. During all three study visits, bleeding events were recorded using the definition of the Bleeding Academic Research Consortium (BARC), which contains unified and validated bleeding criteria [7, 8]. Finally, blood was drawn for extensive testing, including PFTs as described below. At the second visit 6 months post-PCI, we collected information on ischaemic and bleeding events, checked the medication, compliance and side effects. Standard laboratory evaluation during this second visit was performed in the first 200 included patients, and in further patients additional testing was only performed if indicated by clinical clues. If the P2Y12 inhibitor was prescribed for more than 6 months, information on bleeding and ischaemic events was collected during an additional telephone call at 12 months. Thus, depending on duration of combination therapy, the total follow-up time was 6 to 12 months.

### Laboratory evaluation

Information on blood collection and detailed description of all performed laboratory tests is described in the Supplemental data. In short, laboratory evaluation consisted of total blood count, renal function, routine haemostatic parameters, rotational thromboelastometry and thrombin generation assays, and DOAC levels if applicable. On-treatment platelet reactivity was measured using three different platelet function tests with multiple agonists: VerifyNow, Multiple Electrode Impedance Aggregometry by Multiplate, and Light Transmission Aggregometry (LTA). Finally, samples were stored to measure coagulation factors, markers of fibrinolysis, and to perform additional genetic testing (e.g. CYP2C19 polymorphisms).

### Endpoints

The primary endpoint was defined as any bleeding (≥ BARC type 1) according to the Bleeding Academic Research Consortium criteria [7, 8]. The primary ischaemic endpoint was defined as a composite of myocardial infarction [9], ischaemic stroke (including transient ischaemic attack), and all-cause death. Other ischaemic endpoints include coronary revascularisation, peripheral artery disease revascularisation and venous thromboembolism.

### Statistical analysis

Continuous variables are expressed as either mean ± standard deviation for normally distributed traits or median with interquartile range (IQR) otherwise. Categorical variables are expressed as counts and percentages. Statistical analyses were performed with IBM SPSS statistics version 25.0.

## Results

**Fig. 2:**
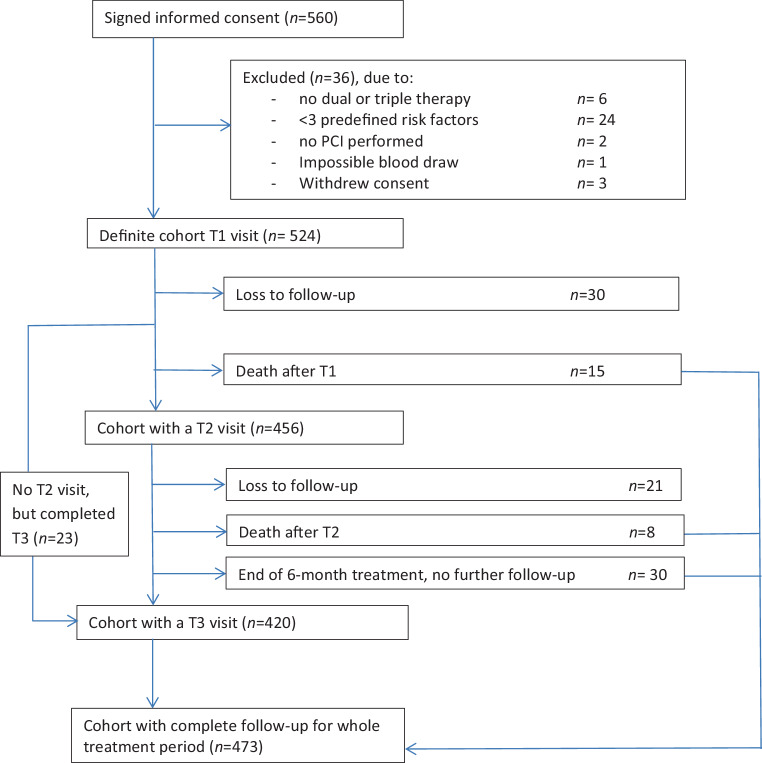
Flowchart of study inclusion and follow-up. *PCI* percutaneous coronary intervention

Initially 560 patients were included in the study and informed consent was obtained. However, subsequently 36 patients had to be excluded for various reasons, and therefore, the final study population consisted of 524 high-risk patients (Fig. 2). Baseline characteristics of the study population are shown in Tab. 2. Mean age is 74.7 ± 8.7 years and patients have a median number of 4 (IQR 3–5) predefined clinical risk factors. At the first study visit (T1), 46 (37–59) days post-PCI, all patients used a P2Y12 inhibitor (clopidogrel *n* = 388, prasugrel *n* = 61, ticagrelor *n* = 75) according to the inclusion criteria, in combination with aspirin (*n* = 392) and/or anticoagulants (*n* = 160). In most patients (*n* = 364, 69.4%) the antithrombotic strategy consisted of dual antiplatelet therapy, whereas 17.0% (*n* = 89) used a P2Y12 inhibitor in combination with anticoagulants, and 13.5% (*n* = 71) had a strategy with triple therapy for at least one month.

**Table 2:** Baseline characteristics of the full cohort (*n* = 524)

Variable	*N* (%), *or *mean ± SD
Age, years	74.7 ± 8.7
Male	302 (57.6)
Body mass index, kg/m^2^ ^a^	27.4 ± 4.6
Current smoking ^b^	72 (13.7)
Alcohol consumption ≥ 7 drinks/week ^c^	99 (18.9)
PPI use at inclusion	441 (84.2)
*Predefined risk factors*	
Number of predefined risk factors, *median [min-max]*	4 [3–9]
– Age ≥ 75 years	318 (60.7)
– Women	222 (42.4)
– Weight ≤ 60 kg	60 (11.5)
– Diabetes mellitus	186 (35.5)
– Hypertension	448 (85.5)
– Anaemia	204 (38.9)
– Renal dysfunction (MDRD-eGFR < 60)	313 (59.7)
– Liver failure	2 (0.4)
– History of gastric/duodenal ulcers	61 (11.6)
– Previous major bleeding	65 (12.4)
– Previous stroke	138 (26.3)
– Use of NSAIDs	21 (4.0)
– Use of SSRIs	31 (5.9)
– Triple antithrombotic therapy	71 (13.5)
– High-risk PCI	47 (9.0)
*Index PCI*	
Acute coronary syndrome	333 (63.5)
Elective procedure	191 (36.5)
Radial access	232 (44.3)
*Number of stents*	
– 0 (DEB, POBA, thrombolysis)	29 (5.5)
– 1	352 (67.2)
– 2	98 (18.7)
– 3	45 (8.6)
*Type of stent/procedure*	
– DES	490 (93.5)
– BMS	4 (0.8)
– Absorb	1 (0.2)
– Drug-eluting balloon	12 (2.3)
– POBA +/− thrombus aspiration	14 (2.7)
– Thrombolysis	3 (0.6)
*Cardiovascular history*	
Prior PCI	197 (37.6)
Prior CABG	106 (20.2)
Prior Stroke	138 (26.3)
Atrial fibrillation	138 (26.3)
Peripheral artery disease	76 (14.5)
Prior venous thromboembolism	39 (7.4)
*Previous history*	
Active malignancy	24 (4.6)
Peptic ulcer disease	61 (11.6)
*Treatment at first study visit*	
P2Y12 inhibitor	524 (100.0)
– Clopidogrel	388 (74.0)
– Prasugrel	61 (11.6)
– Ticagrelor	75 (14.3)
Aspirin	392 (74.8)
Vitamin K antagonist	91 (17.3)
DOAC	68 (13.0)
– Apixaban	20 (3.8)
– Rivaroxaban	34 (6.5)
– Edoxaban	4 (0.8)
– Dabigatran	10 (1.9)
LMWH	1 (0.2)
Dipyridamole	2 (0.4)
*Combination strategies*	
Dual antiplatelet treatment (DAPT)	364 (69.4)
– For 6 months	62 (11.8)
– For 12 months	302 (57.6)
P2Y12 inhibitor with VKA/DOAC/LMWH	89 (17.0)
Initial triple therapy ^f^	71 (13.5)
– For 1 month	64 (12.2)
– For 3–6 months	7 (1.4)
*Laboratory test (reference range)*	*Mean +/− SD*
Haemoglobin	
– Male (8.2–11.0 mmol/l)	8.4 ± 1.1
– Female (7.3–9.7 mmol/l)	8.0 ± 0.9
Haematocrit	
– Male (0.42–0.52 l/l)	0.41 ± 0.05
– Female (0.36–0.48 l/l)	0.39 ± 0.04
MCV (80–100 fl)	91.7 ± 5.8
Platelet count, (150–350 10^9^/l)	261 ± 78
MPV (80–100 fl)	10.3 ± 0.9
PT (9.9–11.5 sec) ^g^	10.7 ± 0.5
APTT (23–32 sec) ^g^	26.2 ± 2.1
Fibrinogen (1.7–4.0 g/l)	3.7 ± 0.9
Creatinine (50–100 µmol/l)	116.6 ± 74.9
MDRD-eGFR (ml/min/1.73 m^2^)	57.1 ± 21.0
*Platelet function test (cut-off values for LPR and HPR *^h^*)*	
Multiplate ADP (19–46 AU) ^d^	47.7 ± 23.2
LTA ADP (20–59% max aggr) ^e^	41.4 ± 16.5
VerifyNow P2Y12 (85–208 PRU) ^a^	136.9 ± 84.7

*PPI *proton pump inhibitor,* MDRD-eGFR *Modification of Diet in Renal Disease—estimated Glomerular Filtration Rate, *NSAIDs *non-steroidal anti-inflammatory drugs,* SSRIs *selective serotonin reuptake inhibitors,* PCI *percutaneous coronary intervention,* DEB *drug-eluting balloon,* POBA *plain old balloon angioplasty,* DES *drug-eluting stent,* BMS *bare metal stent* CABG *coronary artery bypass graft,* DOAC *direct oral anticoagulants,* VKA *vitamin K antagonist,* LMWH *low molecular weight heparin

^a^*missing in 6 patients*; ^b^*missing in 3 patients*; ^c^*missing in 11 patients*; ^d^*missing in 8 patients*; ^e^*missing in 9 patients*; ^f^*Triple therapy consists of a P2Y12 inhibitor plus aspirin plus anticoagulants (VKA, DOAC, LWMH)*; ^g^*in 364 patients not on anticoagulants (VKA, DOAC, LMWH)*; ^h^*cut-off values according to consensus documents *[13–15]

### Follow-up

The second and third study visit took place after a median of 201 days (187–217) and 369 (358–381) days post-PCI respectively. As shown in the flowchart of study inclusion and follow-up (Fig. 2), the cohort of patients with total follow-up for the entire treatment period, or until death as endpoint, consisted of 473 patients (90.3% of the initial cohort).

### Bleeding events

Approximately 1.5 month after PCI (T1), 147 patients (28.1%) reported a total number of 188 bleeding events, 26% of which were BARC type 2 or 3 bleeding events (Tab. 3). Although the prevalence of bleeding symptoms had decreased to 19.5% in the period between T2 and T3 (compared with 28.1% and 29.6% between PCI and T1, and T1 and T2 respectively), the percentage of BARC type 2 or 3 amongst these bleeding events remained stable (29.5% out of 95 bleeding events) as compared with T1.

**Table 3:** Bleeding events and ischaemic events during follow-up

Bleeding endpoint	Cumulative (*n* = 524)	T1 visit (*n* = 524)	T2 visit ^b^ (*n* = 456)	T3 visit ^b^ (*n* = 420)
*Any bleeding*	254 (48.5)	147 (28.1)	135 (29.6)	82 (19.5)
*Most severe bleeding*				
– BARC type 1	162 (30.9)	102 (19.5)	105 (23.0)	54 (12.9)
– BARC type 2	63 (12.0)	34 (6.5)	19 (4.2)	19 (4.5)
– BARC type 3	29 (5.5)	11 (2.1)	11 (2.4)	9 (2.1)
*Total number of bleeding events* ^a^	442	188	159	95
– BARC type 1	332 (75.1)	139 (73.9)	126 (79.2)	67 (70.5)
– BARC type 2	75 (16.9)	36 (19.1)	20 (12.6)	19 (20.0)
– BARC type 3	35 (7.9)	13 (6.9)	13 (8.2)	9 (9.5)
*Ischaemic event*	*Cohort* (*n* *=* *524*)			
No ischaemic events	416 (79.2)			
Major adverse cardiovascular event (myocardial infarction, stroke or all-cause death)	69 (13.2)			
Myocardial infarction	36 (6.9)			
Stent thrombosis	8 (1.5)			
Stroke	13 (2.5)			
Death, all-cause	23 (4.4)			
– Confirmed cardiovascular death ^c^	6 (1.1)			
– Death (non-cardiovascular, unknown)	17 (3.2)			
Coronary revascularisation	37 (7.1)			
PAD with revascularisation	17 (3.2)			
Venous thromboembolism	3 (0.6)			

*BARC* Bleeding Academic Research Consortium*, PAD* peripheral artery disease

^a^ In patients reporting any bleeding symptoms (one patient can report more than one bleeding event at the same visit)

^b^ Bleeding events since last study visit

^c^ Confirmed cardiovascular death is defined as death due to acute myocardial infarction, death due to stroke, or in-hospital cardiac arrest

After 12 months, 254 patients (48.5%) had reported one or more BARC type 1–3 bleeding events. Most patients (30.9%) had only reported mild bleeding (BARC type 1), for which no consultation or interventions were necessary. However, still 92 patients (17.5%) had experienced a BARC type 2 or 3 bleeding at any time point, necessitating consultation, diagnostic tests, interventions, blood transfusions and/or hospitalisation.

### Ischaemic events

During one-year follow-up, 69 patients (13.2%) had a major adverse cardiovascular event (Tab. 3, supplementary Fig. 1); 36 patients with myocardial infarction, 8 patients with confirmed stent thrombosis, 13 patients with stroke and 23 of them had died during follow-up, of whom 6 patients with confirmed cardiovascular death.

### Medication switch

The type, dosage or duration of P2Y12 inhibitor had to be adjusted in 78 patients (14.9%) during 1‑year follow-up due to bleeding episodes, recurrent ischaemic events, risk assessment, PFT results or side effects, or a combination of these. In another 33 patients (6.3%), an unplanned change in anticoagulants and/or aspirin was necessary during follow-up (supplementary Table 1).

### Publications about the cohort to date

In a first publication, the agreement between different platelet function tests, as well as the factors influencing this agreement in vulnerable patients were assessed [10]. Results suggest that the agreement is only slight to moderate, and that PFTs are not interchangeable when determining the response to antiplatelet therapy. More recently, a small study was done focusing on possible strategies to optimise the agreement between the Multiplate and VerifyNow assay [11]. A study on the relationship between genetics (CYP2C19 metabolism) and results of PFTs in clopidogrel-treated patients was presented at the annual meeting of the European Society of Cardiology [12] and the full manuscript is currently in preparation, as well as manuscripts on the value of thrombin generation assays [13] and rotational thromboelastometry. Finally, an interim analysis presented at the Eurothrombosis Congress of the ESC Working Group on Thrombosis showed that using the previously proposed cut-off levels [14–16], PFTs performed at 1 month after PCI were not able to accurately predict bleeding complications in our high-risk population during a 1-year follow-up period [17].

## Discussion

In this paper we present our well characterised cohort of high-risk patients on dual or triple antithrombotic therapy after PCI. This cohort will serve to answer several future research questions about predictors, safety and outcome of patients with multiple clinical risk factors on dual or triple antithrombotic therapy. The high incidence of both bleeding and ischaemic events, as well as the frequent need for medication adjustment during follow-up, indicates the need for strict monitoring of this patient group and illustrates challenges in optimal antithrombotic management.

In the past decade, several studies have shown that tailoring antiplatelet therapy based on PFTs does not prevent ischaemic and bleeding outcomes in the general PCI population [18–20]. With the recent advances in stent technology and broader use of potent P2Y12 inhibitors, thrombotic events have dramatically decreased, and consequently, prevention of bleeding complications has become a major goal [21–23]. Thus, as was also suggested in the recent expert consensus statement on platelet function testing for guiding P2Y12 inhibitor treatment, platelet function testing may play a more important role in a bleeding reduction strategy [22]. Indeed, randomised trials incorporating PFT results to de-escalate DAPT have shown promising results [24, 25]. Reflecting these results, recent guidelines included a Class IIb recommendation for de-escalation of P2Y12 inhibition treatment guided by PFTs to be considered as an alternative DAPT strategy, especially for ACS patients deemed unsuitable for 12-month potent platelet inhibition [1]. Building on this, such a risk assessment strategy might be even more beneficial when results of PFTs are combined with other variables in an algorithm [26]. This cohort can serve to optimise such risk assessment strategies.

### Future directions

To further optimise the applicability of PFTs, adjustment of cut-off levels in various conditions (e.g. type of P2Y12 inhibitor, comorbidities) might be necessary, as the predictive capacity is currently limited. Our data could serve to adjust these cut-off levels for the different PFTs in specific, high-risk patient groups. Furthermore, the descriptive data, in combination with laboratory assays, genetics and bleeding questionnaires could be used for the construction of a multimarker risk prediction model. Current risk prediction models [27–30] are generally developed for the average PCI population, whereas a risk prediction model specifically developed for a high bleeding risk population currently does not exist [5]. At a later stage, such a model could be used in intervention studies stratifying therapy to high-risk patients. Collaboration with other research groups with comparable data is welcomed, and would be beneficial to further the prediction modelling plans. Besides optimisation of the combination and treatment duration of antithrombotic therapy in high-risk patients, new treatment options for high-risk patient populations are on the way. These recent advances not only involve new antithrombotic strategies (e.g. dual pathway inhibition [31]), but also anti-inflammatory drugs (e.g. canakinumab [32] or colchicine [33, 34]). These new therapies could be implemented and evaluated when continuing data collection on future cohorts of comparable high-risk patients in our centre.

### Strengths and limitations

Strengths of this study are that it comprises a large prospective clinical cohort with detailed data and extensive laboratory testing. Particularly valuable is the comparison of three different PFTs with multiple agonists in a large cohort of high-risk patients. Another strength of our study is the detailed information on minimal bleeding events (BARC type 1), which were collected during the whole follow-up, although retrospectively from PCI until the first study visit. These minimal bleeding events often have an impact on patients’ daily life, but as most studies only collect the bleeding events retrospectively, these BARC type 1 bleeding events could often not be reported. A limitation of our study is that due to rapid developments in stent technology, stronger platelet inhibition and guideline updates, the relatively long inclusion time of 5 years may have caused heterogeneity within the cohort. Moreover, due to the observational nature of the study, some patients decided to refrain from further hospital visits, chose to visit their regional cardiologist or general practitioner instead, or could not be contacted for study visits, leading to loss to follow-up in 9.7%. Another limitation might be that the identification of risk factors for selection of high-risk patients was based on literature and expert consensus when initiating the study in 2014. Only recently, a consensus document from the Academic Research Consortium for High Bleeding Risk (ARC-HBR) was published, presenting a consensus definition of patients at high bleeding risk [5]. Our risk factors show substantial overlap with this consensus definition; all minor criteria were included and out of the major criteria only recent or non-deferrable major surgery was not counted as a risk factor for inclusion in this cohort study. However, these data are retrievable when needed for analysis. Data on thrombocytopenia, active malignancy and chronic bleeding diathesis were structurally collected but not counted as a predefined risk factor in our cohort. In fact, due to concurrent research on platelet function and clotting factors, thrombocyte count < 100 and known coagulation disorders were exclusion criteria in our study. However, the most important and reliable predictor of bleeding in patients with bleeding diatheses is a personal history of bleeding, which can be assessed with a bleeding questionnaire [5, 35], and this valuable information was collected in our study.

## Conclusion

In this well characterised cohort of patients with multiple clinical risk factors treated with dual or triple antithrombotic therapy after PCI, we showed the high risk for both bleeding and ischaemic events. This challenges the treating physician to make a balanced decision on the optimal, individualised antithrombotic treatment strategy. Future results of this cohort study will serve to further expand the knowledge on the optimal treatment of these high-risk patients, and the implementation of patient characteristics and a wide range of laboratory tests to guide treatment decisions.

## Supplementary Information

Supplemental data: detailed information on methods of blood collection, laboratory evaluation and platelet function tests

*Supplementary Fig. 1: *Kaplan-Meier curve for ischaemic events. Event-free survival for major adverse cardiovascular events (a composite of myocardial infarction, ischaemic stroke and all-cause death) during follow-up.

*Supplementary Table 1:* Medication switches during follow-up in full cohort (*n* = 524)
