# Supplementary material for: Antithrombotic therapy in high-risk patients after percutaneous coronary intervention; study design, cohort profile and incidence of adverse events
Source: Neth Heart J. 2021 Sep 1;29(10):525–35. doi: 10.1007/s12471-021-01606-2 (PMC8455732; doi:10.1007/s12471-021-01606-2)
Supplement: Supplementary file 1 — Supplemental data: detailed information on methods of blood collection, laboratory evaluation and platelet function tests [file 12471_2021_1606_MOESM1_ESM.docx]

*Supplemental data*

*Laboratory evaluation*

Patients were asked to avoid fat-rich food 4 hours prior to blood withdrawal. Venous blood was collected by antecubital venepuncture on the same day as the first outpatient visit. Standard laboratory evaluation consisted of total blood count (Sysmex, XN-9000), renal function (Cobas-8000; Roche reagents), and routine haemostatic parameters (Sysmex CS-2100; Siemens reagents). In patients using direct oral anticoagulants (DOAC), the DOAC levels were measured using specific anti-Xa levels for direct factor Xa-inhibitors (Biophen, daXa), or diluted thrombin time (dTT) for patients using dabigatran (Biophen, Hemoclot®). On-treatment platelet reactivity was measured using three different platelet function tests (PFTs), as described below. Additionally, thrombin generation assays (Calibrated Automated Thrombography, CAT) and rotational thromboelastometry (Werfen, ROTEM® delta) were performed. Finally, samples were stored to measure coagulation factors, von Willebrand factor (vWF), markers of fibrinolysis, and to perform additional genetic testing (e.g. CYP2C19 polymorphisms). Platelet function measurements were started within one hour of blood withdrawal, which was done simultaneously for the following three assays.

Multiple electrode impedance Aggregometry by Multiplate

Blood was collected in Hirudin Blood Tubes (3mL, Double Wall; Verum Diagnostica GmbH, Munich). Agonist-induced platelet aggregation was measured by the Multiplate Analyser (Dynabyte, Munich, Germany), according to manufacturer’s instructions. The Multiplate analyser is a multiple electrode impedance aggregometer that measures platelet aggregation in whole blood. Agonists used were adenosine diphosphate (ADP) (6.4 μmol/L; Roche), Collagen (3.2 ug/mL), thrombin receptor-activating peptide (TRAP) (32 µmol/L; Roche) and arachidonic acid (AA) (0.5mM; Roche). Results are expressed as arbitrary aggregation units (AU). For evaluation of P2Y12 receptor blockers, the ADP-induced platelet aggregation was used. According to a previous consensus document by Tantry et. al, low on-treatment platelet reactivity is defined as AU <19 and high on-treatment platelet reactivity as AU >46 [1].

VerifyNow

Blood was collected in 3.2% sodium citrate Vacuette partial-fill tubes (2mL; Greiner Bio-One, GmbH, Kremsmuntster, Austria). VerifyNow ASPI and VerifyNow P2Y12 assay (Accumetrics Inc, San Diego, CA, USA) were performed according to manufacturer’s instructions. VerifyNow is a turbidimetric-based optical detection system, in which whole blood is added to a device using fibrinogen-coated microbeads and, after addition of an agonist, the increase in light transmittance is detected to measure platelet reactivity. Agonists used were AA (1 mmol/L; Accumetrics), or, for evaluation of P2Y12 inhibitors, a combination of ADP (20 µmol/L) and prostaglandin E1 (22nmol/L). Results are expressed in P2Y12 reaction units (PRU), with low on-treatment platelet reactivity being defined as PRU <85 and high on-treatment platelet reactivity as PRU >208 [1].

### Light Transmission Aggregometry (LTA)

Blood was collected in 3.2% sodium citrate Vacuette tubes (9mL; Greiner Bio-One). For preparation of platelet-rich plasma, blood was centrifuged at 170 g for 10 min at 18 °C. For preparation of platelet-poor plasma citrated-blood was centrifuged at 2500 g for 5 min and then at 10000 g for 10 min at 18 °C. Platelet count in platelet-rich plasma was adjusted with autologous platelet-poor plasma to 250 x 10^9^ platelets/L. In vitro platelet aggregation was measured in response to AA (1mM; Bio Date Corporation (Emergo)), ADP (20 μmol/L; Chrono-Par, CH 384), and TRAP (15 µmol/L; Boom H8105) at 37 °C (Chrono-log 490-4D; Chrono-Log Corp.). For evaluation of P2Y12 inhibitors, the main result was the percentage of maximal platelet aggregation in response to ADP. Low on-treatment platelet reactivity was defined as maximal aggregation <20% [2] and high on-treatment platelet reactivity as maximal aggregation >59% [3].

1. Tantry, U.S., et al., *Consensus and update on the definition of on-treatment platelet reactivity to adenosine diphosphate associated with ischemia and bleeding.* J Am Coll Cardiol, 2013. **62**(24): p. 2261-73.

2. Kerneis, M., et al., *Switching acute coronary syndrome patients from prasugrel to clopidogrel.* JACC Cardiovasc Interv, 2013. **6**(2): p. 158-65.

3. Bonello, L., et al., *Consensus and future directions on the definition of high on-treatment platelet reactivity to adenosine diphosphate.* J Am Coll Cardiol, 2010. **56**(12): p. 919-33.
