# Supplementary figures and images for: Antithrombotic therapy in high-risk patients after percutaneous coronary intervention; study design, cohort profile and incidence of adverse events
Source: Neth Heart J. 2021 Sep 1;29(10):525–35. doi: 10.1007/s12471-021-01606-2 (PMC8455732; doi:10.1007/s12471-021-01606-2)

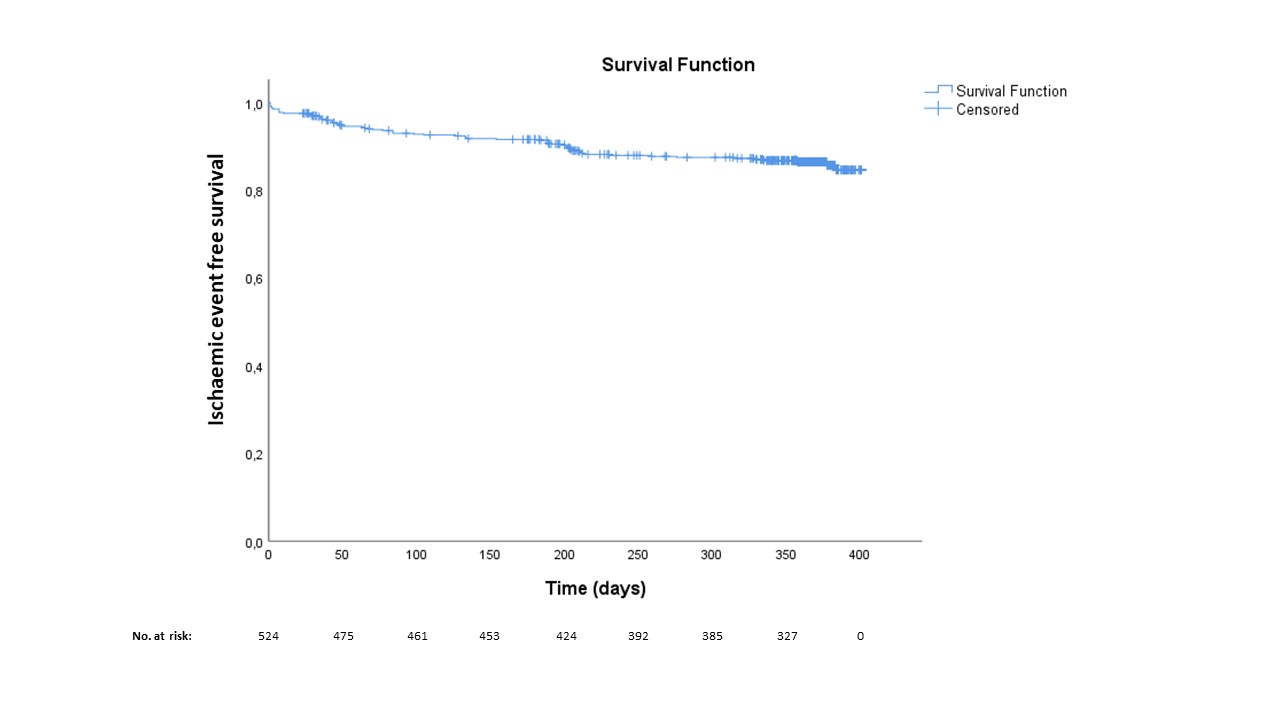

Supplement: Supplementary file 2 — Supplementary Fig. 1: Kaplan-Meier curve for ischaemic events. Event-free survival for major adverse cardiovascular events (a composite of myocardial infarction, ischaemic stroke and all-cause death) during follow-up. [file 12471_2021_1606_MOESM2_ESM.docx]
