# Supplementary material for: Antithrombotic therapy in high-risk patients after percutaneous coronary intervention; study design, cohort profile and incidence of adverse events
Source: Neth Heart J. 2021 Sep 1;29(10):525–35. doi: 10.1007/s12471-021-01606-2 (PMC8455732; doi:10.1007/s12471-021-01606-2)
Supplement: Supplementary file 3 — Supplementary Table 1: Medication switches during follow-up in full cohort (n = 524) [file 12471_2021_1606_MOESM3_ESM.docx]

Supplementary Table 1: Medication switches during follow-up in full cohort (*n*=524)

| **Treatment** | **Type of adjustment** | **(de-)escalation** | **Adjustment of therapy** | ***N*** |
| --- | --- | --- | --- | --- |
| **P2Y12 inhibitor**  ***n*=78** | Medication type  *n*=68 | Escalation  *n*=26 | Clopidogrel – Prasugrel  Clopidogrel – Ticagrelor  Prasugrel 5 mg – 10 mg | 18  8  0 |
|  |  | De-escalation *n*=37 | Ticagrelor – Clopidogrel  Prasugrel – Clopidogrel  Prasugrel 10 mg – 5 mg | 18  10  9 |
|  |  | Other  *n*=5 | Ticagrelor – Prasugrel  Prasugrel – Ticagrelor  Other # | 1  2  2 |
|  | Duration  *n*=10 | | Shortened | 9 |
|  |  |  | Prolonged | 1 |
|  | | | | |
| **Aspirin *n*=10** | Start | | | 3 |
|  | Prematurely stopped | | | 7 |
|  | | | | |
| **Oral anticoagulants**  ***n*=23** | Type OAC *n*=6 | | VKA - DOAC | 1 |
|  |  |  | DOAC - VKA | 1 |
|  |  |  | DOAC - DOAC | 4 |
|  | Dose adjustment *n*=8 | | Escalation | 1 |
|  |  |  | De-escalation | 7 |
|  | Start | | | 8 |
|  | Stop | | | 1 |

# Patients accidentally used ticagrelor once daily instead of twice daily

Abbreviations: OAC, oral anticoagulants; VKA, vitamin K antagonist; DOAC, direct-acting oral anticoagulants
